# Small Intestine Early Innate Immunity Response during Intestinal Colonization by *Escherichia coli* Depends on Its Extra-Intestinal Virulence Status

**DOI:** 10.1371/journal.pone.0153034

**Published:** 2016-04-20

**Authors:** Jérôme Tourret, Benjamin P. Willing, Matthew A. Croxen, Nicolas Dufour, Sara Dion, Sarah Wachtel, Erick Denamur, B. Brett Finlay

**Affiliations:** 1 IAME, UMR 1137, INSERM, Paris, France; 2 Univ. Paris Diderot, Sorbonne Paris Cité, Paris, France; 3 AP-HP, Groupe Hospitalier Universitaire Pitié Salpêtrière Charles Foix, Département d’urologie, néphrologie et transplantation, Paris, France; 4 Univ. Pierre et Marie Curie, Sorbonne Universités, Paris, France; 5 Michael Smith Laboratories, The University of British Columbia, Vancouver, Canada; 6 Department of Agricultural, Food and Nutritional Science, University of Alberta, Edmonton, Alberta, Canada; 7 British Columbia Centre for Disease Control Public Health Laboratory, Vancouver, British Columbia, Canada; 8 Lurie Children's Hospital of Chicago Research Center, Department of pediatrics, Northwestern University Feinberg School of Medicine, Chicago, United States of America; 9 Institut Pasteur, Molecular Biology of Gene in Extremophiles, Department of Microbiology, Paris, France; 10 AP-HP, Hôpital Louis Mourier, Service de Réanimation Médico-chirurgicale, Colombes, France; Université d'Auvergne Clermont 1, FRANCE

## Abstract

Uropathogenic *Escherichia coli* (UPEC) strains live as commensals in the digestive tract of the host, but they can also initiate urinary tract infections. The aim of this work was to determine how a host detects the presence of a new UPEC strain in the digestive tract. Mice were orally challenged with UPEC strains 536 and CFT073, non-pathogenic strain K12 MG1655, and ΔPAI-536, an isogenic mutant of strain 536 lacking all 7 pathogenicity islands whose virulence is drastically attenuated. Intestinal colonization was measured, and cytokine expression was determined in various organs recovered from mice after oral challenge. UPEC strain 536 efficiently colonized the mouse digestive tract, and prior Enterobacteriaceae colonization was found to impact strain 536 colonization efficiency. An innate immune response, detected as the production of TNFα, IL-6 and IL-10 cytokines, was activated in the ileum 48 hours after oral challenge with strain 536, and returned to baseline within 8 days, without a drop in fecal pathogen load. Although inflammation was detected in the ileum, histology was normal at the time of cytokine peak. Comparison of cytokine secretion 48h after oral gavage with *E*. *coli* strain 536, CFT073, MG1655 or ΔPAI-536 showed that inflammation was more pronounced with UPECs than with non-pathogenic or attenuated strains. Pathogenicity islands also seemed to be involved in host detection, as IL-6 intestinal secretion was increased after administration of *E*. *coli* strain 536, but not after administration of ΔPAI-536. In conclusion, UPEC colonization of the mouse digestive tract activates acute phase inflammatory cytokine secretion but does not trigger any pathological changes, illustrating the opportunistic nature of UPECs. This digestive tract colonization model will be useful for studying the factors controlling the switch from commensalism to pathogenicity.

## Introduction

Urinary tract infections (UTIs) are among the most common infections worldwide, affecting one in two women [1]. *Escherichia coli* is responsible for 50–90% of all UTIs [2].

Classically, three major *E*. *coli* phenotypes are distinguished: non-pathogenic *E*. *coli*, intestinal pathogenic (InPEC) and extra-intestinal pathogenic *E*. *coli* (ExPEC) [3]. Non-pathogenic *E*. *coli* strains show no virulence ability in any animal model, and are not usually isolated outside of the gastrointestinal tract from sick immunocompetent patients. They also express no or very few virulence factors. InPECs are responsible for several types of diarrhea [4], and are currently a leading cause of mortality in children worldwide [5]. ExPECs are responsible for diseases outside of the digestive tract, including urinary tract infections (due to the subgroup of uropathogenic *E*. *coli* (UPEC) strains), newborn meningitis, visceral abscesses, and septicemia [3]. Interestingly, this observational classification has evolutionary foundations [6]. Virulence is linked to phylogeny in *E*. *coli* species, with ExPEC predominantly belonging to phylogenetic groups B2 and D, while commensal and InPEC are found mainly in groups A and B1.

However, the boundaries between these phenotypic groups are often tenuous. For example, non-pathogenic phylogroup A *E*. *coli* strains have been isolated from bloodstream infections of immunocompromised patients [7]. The Shiga toxin-producing *E*. *coli* (STEC) strain O157:H7 is a non-pathogenic commensal of cattle [8], but causes bloody diarrhea in humans (for this reason it is considered an InPEC) [9], and has been isolated from urinary tract infections in patients with or without uremic and hemolytic syndrome (but not frequently enough to be classified within the ExPEC) [10, 11].

One common characteristic of all *E*. *coli* strains is that their primary habitat is the lower digestive tract of vertebrates. It has been suspected for several decades [12, 13]–and now, well documented [14]–that before reaching extra-intestinal sites, such as the urinary tract, ExPEC first must integrate as part of the gut microbiota. Some individuals harbor potentially extra-intestinal virulent *E*. *coli* strains in their digestive tract, and are therefore considered “healthy carriers” of ExPEC [15]. These strains often belong to B2 or D phylogenetic groups, can express many virulence factors, and are lethal when injected subcutaneously to mice [16]. Whether and when these intrinsically virulent commensal strains are going to be responsible for an extra-intestinal infection remains unknown, as no prospective study on extra-intestinal infection kinetics has been reported. Worldwide spread of clonal group A, which belongs to phylogroup D, is an illustration of this continual alternation between a commensal life in a healthy colon and an extra-intestinal pathogenic phase; it has been isolated from the urine of women suffering from community acquired UTI in the US [17, 18], from the blood of septicaemic patients in Europe [19], and from the feces of healthy individuals in several locations in the US [20]. An oro-fecal mode of transmission has even been suggested in some community UTI outbreaks [17].

Assuming ExPEC can lead a commensal life, the determinants of the commensalism-to-pathogenicity switch remain largely unknown. Understanding the factors (depending on the host, the bacterium and the environment) that trigger an extra-intestinal infection in a healthy ExPEC carrier is of importance as it could lead to new preventive strategies.

Based on these observations, it reasons that intestinal colonization is the first step of any extra-intestinal infection. Therefore, allowing a potentially pathogenic strain to colonize the gut is a potential threat for the host, while keeping a pathogen-free intestine ensures subsequent health.

The main goal of this work was to determine how the establishment of a UPEC strain as a commensal in the digestive tract was detected by the host, and whether the detection was in relation to virulence status of the incoming strain. To address these questions, we developed a new mouse model to study digestive tract colonization by *E*. *coli*. This model did not involve antibiotic treatment and thereby avoided disruption of the resident microbiota, as antibiotic-induced alterations in the gut microbiota influence host immunity [21, 22].

## Material and Methods

### Bacterial strains

Uropathogenic *E*. *coli* strain 536 was isolated from the urine of a German patient suffering from acute pyelonephritis [23]. It belongs to the B2 phylogenetic group, sub-group III, STc127 [24], and harbors an O6 serogroup [25]. Its genome has been completely sequenced [26]. This strain is naturally resistant to streptomycin.

ΔPAI-536 is an isogenic mutant of *E*. *coli* strain 536 in which all 7 pathogenicity islands have been deleted [27]. The virulence of this mutant is drastically decreased [28].

Strain K-12 MG1655 is a laboratory-derived commensal *E*. *coli* strain. It belongs to the A phylogenetic group, harbors serogroup O16 and has no antibiotic resistance or virulence factors. Strain CFT073 is a uropathogenic *E*. *coli* strain [29], that belongs to the B2 phylogenetic group, sub-group II, STc73 and harbors serogroup O6.

### Ethics statement

All animal experiments were performed in strict accordance with the guidelines of the University of British Columbia Animal Care Committee and the Canadian Council on the Use of Laboratory Animals. The protocol was approved by the UBC Animal Care Committee (Certificate number: A09-0168). Mice were monitored once daily by the experimenter and once daily by the animal facility staff throughout the experiment. An early endpoint (weight loss >15%) was used to euthanize severely ill animals prior to the experimental endpoint. No animal died prior to the experimental endpoint, and no animal ever reached the early endpoint. The mice were euthanized by CO_2_ asphyxiation and all efforts were made to minimize suffering.

### Mice

All experiments were performed on 6-week old C3H/HeOuJ or C57BL/6J mice (Jackson Laboratory, Bar Harbor, ME, specific and opportunistic pathogen free upon arrival), housed in the specific pathogen-free animal facility at the University of British Columbia (UBC). Mice were fed standard chow (Laboratory Rodent Diet 5001, Purina Mills, St. Louis, Missouri) *ad libitum* throughout experiments, and had free access to sterile water. Prior to any treatment, one fecal pellet (minimum weight: 20 mg) was collected from each mouse. Fecal samples were resuspended in 1 mL of sterile phosphate-buffered saline (PBS) with a MM 301 mixer mill (Retsch, Newtown, PA), for 3 min at 25 Hz, and 100 μL of the fecal suspension were plated on MacConkey and on MacConkey+streptomycin (30 μg/mL) agar plates. The limit of detection was 500 CFU/g of feces, i.e. 2,7 log_10_ (CFU/g feces). All C57BL6/J mice were free of Enterobacteriaceae (no growth on MacConkey agar upon arrival and until the day mice were used for oral gavage). In contrast upon arrival, some cages harbored C3H/HeOuJ mice who all contained Enterobacteriaceae, while other cages harbored only Enterobacteriaceae-free C3H/HeOuJ mice. However, no C3H/HeOuJ mice contained streptomycin-resistant Enterobacteriaceae upon arrival in the mouse facility and until mice were used for oral gavage.

### *E*. *coli* oral challenge

A single colony of *E*. *coli* strains 536, ΔPAI-536, MG1655 or CFT073 was inoculated in 5 ml of lysogeny broth at 37°C, overnight under constant shaking (200 rpm). The following day, 1 mL of culture was spun down (6 min at 6000 x g), and washed twice in PBS. Mice received oral gavage with a 100 μL suspension containing 10^7^ colony forming units (CFU). For each experiment, the inoculum was checked by plating serial dilutions on MacConkey agar plates. *E*. *coli* strain 536 was indifferently administered to mice colonized with or free of Enterobacteriaceae, whereas Enterobacteriaceae-free mice only were used to administer ΔPAI-536, MG1655 or CFT073. Fecal pellets were collected every day or every other day after mice were individually placed in isopropanol-washed plastic jars. After weighing, a small fecal sample was resuspended in 1 mL of PBS. Ten-fold serial dilutions were plated on MacConkey (MG1655 or CFT073) or on MacConkey+streptomycin 30 μg/mL (*E*. *coli* strain 536 and ΔPAI-536) agar plates. Each experiment was repeated at least twice, with at least 4 mice in each tested condition.

### Organ supernatant preparation

Mice were euthanized by cervical dislocation following CO_2_ asphyxiation at the desired time point. Bladders, kidneys, spleens, livers, and mesenteric lymph nodes were collected in 250 μL– 1000 μL of PBS + 2X Complete^®^ EDTA-free proteinase inhibitor (Roche^™^, 1 tablet for 25 mL of PBS). Segments of ileum, caecum and colon were collected in 1 mL of PBS + 2X Complete^®^ EDTA-free proteinase inhibitor + 0.01% soybean trypsin inhibitor (Sigma). Upon collection, all samples were kept at 4°C throughout sample processing, and subsequently frozen at -80°C. All organs were collected in 2 mL safe-lock^®^ tubes containing one autoclaved 5 mm tungsten bead. Samples were resuspended by shaking (mixer-mill, 3 min, 25 Hz), and centrifuged (5 min 14,000 g). Supernatants were transferred to fresh tubes and frozen at -80°C.

### Simultaneous multiple cytokine measurements in organ supernatants

Monocyte Chemotactic Protein 1 (MCP-1), Interleukin (IL) 1β, IL-4, IL-5, IL-6, IL-10, IL-12, IL-13, IL-17, IL-21, Interferon (IFN) γ, and Tumor Necrosis Factor (TNF) α were simoultaneously measured in multiple samples using BD bioscience^™^ Cytometric Bead Array (CBA flex set multiplex) according to manufacturer’s instructions. Organ supernatants were thawed at 4°C, and 50 μL were used for the cytokine screen. Cytokine concentrations were normalized by the sample weight.

### ELISA

Organ supernatants were thawed at 4°C, and 100 μL were used for IL-6, IL-10, and TNF-α enzyme-linked immunosorbent assays (ELISAs) (OptEIA^®^, BD biosciences^™^) according to manufacturer’s instructions. Cytokine concentrations were normalized by the sample weight.

### Histopathology

Ileum samples were fixed in 10% neutral buffered formalin overnight and then placed in 75% ethanol. Fixed tissues were embedded in paraffin and cut into 5 μm sections. Tissues were stained with haematoxylin and eosin, using standard techniques by Wax-it Histology Services (Vancouver, BC, Canada).

### Statistical analyses

Series of homoscedastic data (Brown-Forsythe’s and Barlett’s tests) with a normal distribution (Kolmogorov-Smirnov test) are expressed as means ± standard deviation and compared by t-tests or one-way ANOVA with Bonferroni’s correction for multiple comparisons, as appropriate. Series of non-Gaussian data are represented as median and interquartile range or box and whiskers (median, interquartile range and minimum-maximum values), and compared by Mann-Whitney U test or Kruskal-Wallis test with Dunn’s correction for multiple comparisons, as appropriate. The proportion of mice who cleared *E*. *coli* were compared by Χ² test. The test that was used is specified in the result section and in the legend of each figure.

All statistics were performed using Prism^®^ 6.05 (GraphPad Software Inc).

## Results

### UPEC strain 536 efficiently colonizes the mouse digestive tract

An inoculum of 10^7^ CFU of *E*. *coli* strain 536 was orally administered to C3H/HeOuJ mice and intestinal colonization was assessed by plating fecal samples on MacConkey+streptomycin (30 μg/mL) agar plates daily or every second day for 8 days. *E*. *coli* strain 536 is naturally resistant to streptomycin, facilitating selection. Mice were free of streptomycin resistant Enterobacteriaceae upon arrival in the animal facility (see Material and Methods). Additionally, 50 fecal clones were randomly selected on MacConkey plates and tested for *E*. *coli* phylogenetic group and B2 sub-group by quadriplex and specific PCR, respectively [30, 31]. Only B2 sub-group III clones were isolated, which corresponds to *E*. *coli* strain 536 group and sub-group.

**Fig 1:**
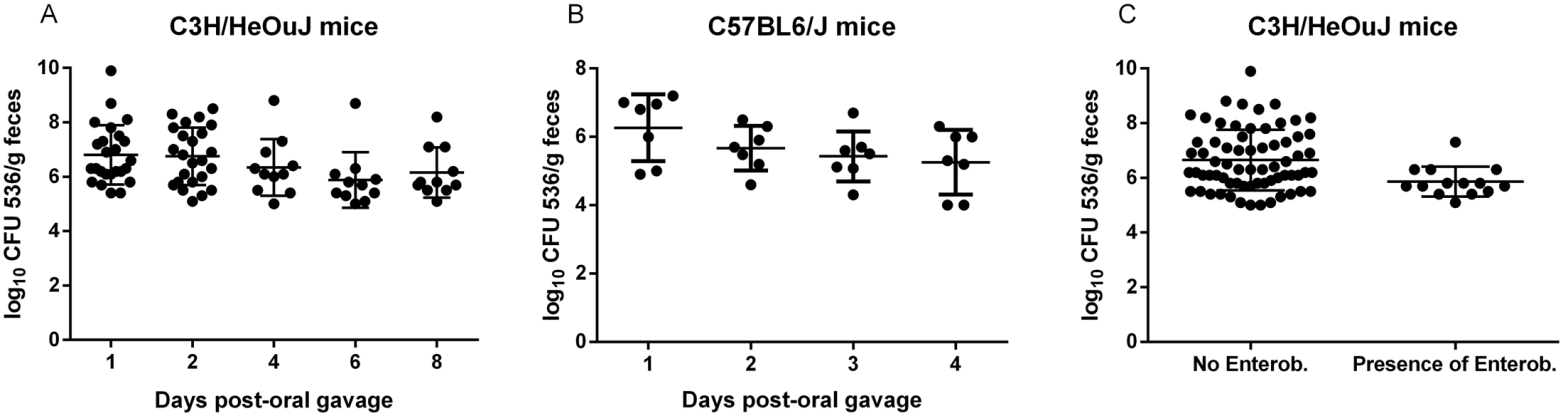
Intestinal colonization after a single oral challenge with 10^7^ CFU of UPEC strain 536. Fecal pellets were individually collected from C3H/HeOuJ (A) or C57BL6/J (B) mice every day or every other day after oral challenge. *E*. *coli* strain 536 CFUs were enumerated in the feces by serial dilution on MacConkey+streptomycin (30 μg/mL) agar plates and expressed as log_10_ (CFU/g feces). Mean bacterial loads were not significantly different over time (Fig 1A and 1B, one-way ANOVA with Bonferroni’s correction for multiple comparisons). C: Some C3H/HeOuJ mice provided by Jackson Laboratory were colonized by Enterobacteriaceae and others were not. Here, fecal counts on days 1 to 8, from Fig 1A are pooled, and represented according to the presence or absence of Enterobacteriaceae prior to oral gavage with *E*. *coli* strain 536. The line indicates a significant difference with p<0.01 (t-test). Bars and whiskers represent means ± standard deviation (Fig 1A and B) or median and interquartile range (Fig 1C).

After a single oral challenge, inter-individual digestive tract colonization was variable but high, ranging from 5.0 to 9.9 log_10_ (CFU/g of feces) (Fig 1A). The mean bacterial count was 6.9±1.1 log_10_ (CFU/g of feces) and remained greater than 5.9 log_10_ (CFU/g of feces) throughout the experiment. The mean fecal concentration of *E*. *coli* was not statistically different from day 1 through day 8 after oral challenge (p = 0.1 by one-way ANOVA), indicating stable colonization on the short term. Interestingly, each mouse maintained their initial (day 1) level of *E*. *coli* throughout the experiment (until day 8), i.e. mice with the highest/lowest *E*. *coli* fecal burden kept a high/low burden throughout the experiment. Colonization of caecum and ileum paralleled fecal concentrations (data not shown).

Colonized mice showed no overt sign of disease. They did not suffer from diarrhea, and their weight remained stable throughout the experiment (data not shown).

In order to ensure that *E*. *coli* strain 536 colonization capacity was not restricted to C3H/HeOuJ mice, the same inoculum was orally administered to C57BL6/J mice. Similar colonization properties were observed (Fig 1B). Mean fecal concentration in C57BL6/J mice did not significantly decrease during the first four days after oral gavage (p = 0.15 by one-way ANOVA). As the purpose of this work was to study the very initial phase of intestinal colonization, fecal bacterial concentration in C57BL6/J mice was not assessed after day 4.

### Pre-colonization with Enterobacteriaceae drastically affects subsequent *E*. *coli* strain 536 intestinal colonization

Upon arrival at the animal facility, a fecal sample was taken from each mouse and plated on MacConkey agar. Some C3H/HeOuJ mice showed intestinal colonization with Enterobacteriaceae (possibly non-*pneumoniae Klebsiella*, as indicated by the provider). Others were free of Enterobacteriaceae based on plating on MacConkey agar. However, no mice showed intestinal colonization with streptomycin-resistant Enterobacteriaceae as indicated by the absence of bacterial growth on MacConkey+streptomycin agar plates. Fig 1C shows pooled *E*. *coli* fecal counts from day 1 to day 8 post gavage as a function of Enterobacteriaceae pre-colonization. Enterobacteriaceae-colonized mice showed significantly lower colonization over 8 days after a single oral challenge than Enterobacteriaceae-free mice. Median fecal bacterial count of Enterobacteriaceae-free mice was 6.3 log_10_ (CFU/g of feces) while median fecal bacterial count of Enterobacteriaceae-colonized mice was 5.7 log_10_ (CFU/g of feces)(p<0.01 by t-test). This indicates that intestinal colonization by an incoming strain can be affected by resident phylogenetically related bacteria.

### *E*. *coli* strain 536 establishment in the mouse digestive tract triggers an innate immunity response in the ileum

**Fig 2:**
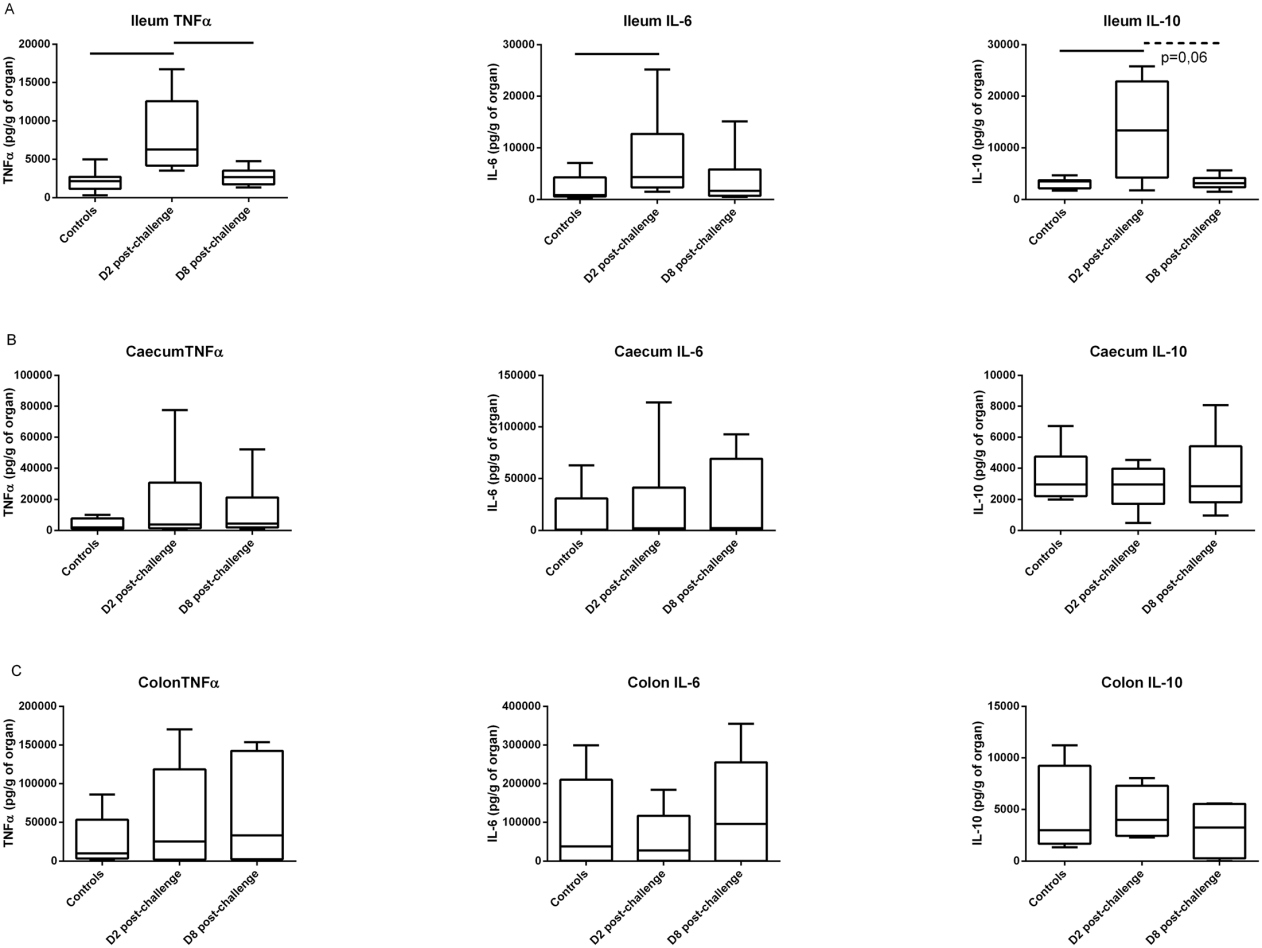
TNFα, IL-6, and IL-10 cytokine ELISA measurements in the ileum (A), caecum (B) and colon (C) 2 days after oral gavage with 100 μL of PBS (controls), and 2 or 8 days after oral gavage with 10^7^ CFU of *E*. *coli* strain 536. Box and whiskers represent medians and interquartile ranges. Lines indicate significant differences with p<0.01 (Mann-Whitney U test).

To determine whether intestinal colonization of a UPEC strain was detected by the host through inflammation, we measured cytokine production. We used a cytometric bead assay screen to simultaneously measure expression of 13 cytokines in 7 organs of 3 groups of 4 mice. The measured cytokines were: MCP-1, IL-1β, IL-12, IL-6, TNFα (in order to explore acute phase and innate immunity), IFNγ, (and again TNFα, Th1 inflammatory response), IL-4, IL-5, IL-6, IL-13 (Th2 inflammatory response), IL-17, IL-21 (Th17 inflammatory response) and IL-10 (Treg activation). These cytokines were measured in homogenates of bladder, spleen, kidney, mesenteric lymph nodes, ileum, caecum, and distal colon. Cytokines were measured in mice sacrificed 2 or 8 days after an oral challenge with *E*. *coli* strain 536 or PBS (controls). This screen showed elevated levels of IL-6, TNFα and IL-10 secretion in the ileum 2 days after oral challenge, but not of the other cytokines (data not shown). In order to confirm this result, specific ELISAs were performed on supernatants of ileum, colon or caecum homogenates (Fig 2). Control mice were sacrificed 2 days after PBS oral gavage, while test mice were sacrificed either 2 or 8 days after a single oral challenge with 10^7^ CFU *E*. *coli* strain 536. At day 2, median TNFα, IL-6, and IL-10 concentrations increased 2.9, 5.0, and 3.8 fold respectively, in the ileum of *E*. *coli*-challenged mice compared to control mice. By day 8 post-infection, cytokine secretion in the ileum returned to a level that was not statistically different from control mice (Fig 2A). In contrast, TNFα, IL-6 and IL-10 secretion was indistinguishable between orally-challenged and control animals at day 2 in the caecum and the distal colon (Fig 2B and 2C).

The cytokine secretion profile and timing is consistent with an acute phase inflammatory response in the ileum. Because no inflammatory response was found in any other portion of the intestine, the ileum may be the specific site of recognition of this potentially harmful extra-intestinal pathogen after oral ingestion.

### *E*. *coli* strain 536 does not induce enteritis after oral challenge

**Fig 3:**
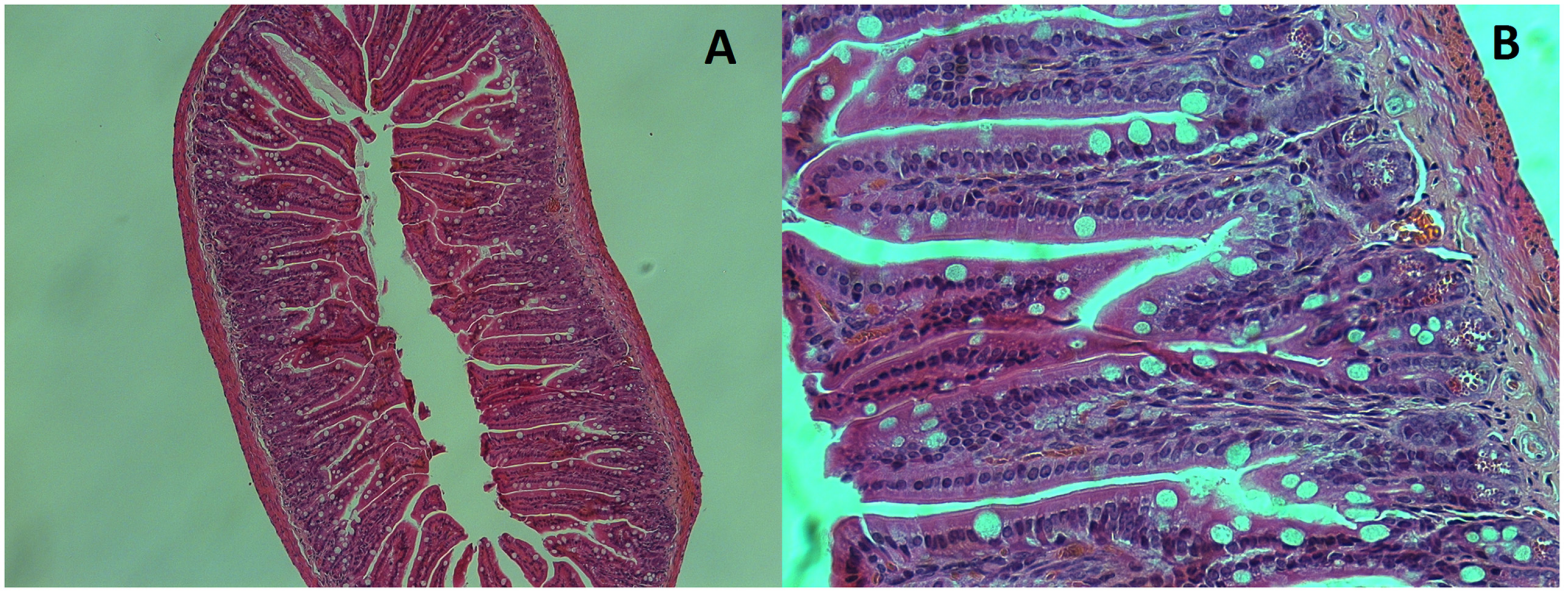
A, and B: Hematoxylin and eosin stain of an ileum section of a mouse 2 days after oral challenge with 10^7^ CFU of *E*. *coli* strain 536. A: 10X magnification. B: 40X magnification.

Because acute phase cytokines were detected in the ileum 2 days after oral challenge with *E*. *coli* strain 536, histologic analysis was performed to look for enteritis. Hematoxylin and eosin stains were performed on ileum sections of mice sacrificed 2 days after oral challenge. These sections showed no sign of inflammation (Fig 3). There was no detectable degree of mucosal edema or hemorrhage, leucocyte infiltrate, intestinal epithelial cell necrosis, or epithelial cell debris shedding in the lumen. Crypt organization was always conserved. All the sections were optically normal.

This indicates that even though *E*. *coli* strain 536 elicits the production of inflammatory cytokines in the ileum, this inflammation remains subclinical. Thus *E*. *coli* strain 536 is an extra-intestinal pathogen that is not capable of inducing clinical enteritis in this model.

### UPEC strains 536 and CFT073 induce a more pronounced ileal innate immunity response than non-pathogenic *E*. *coli* strains MG1655 and ΔPAI-536

**Fig 4:**
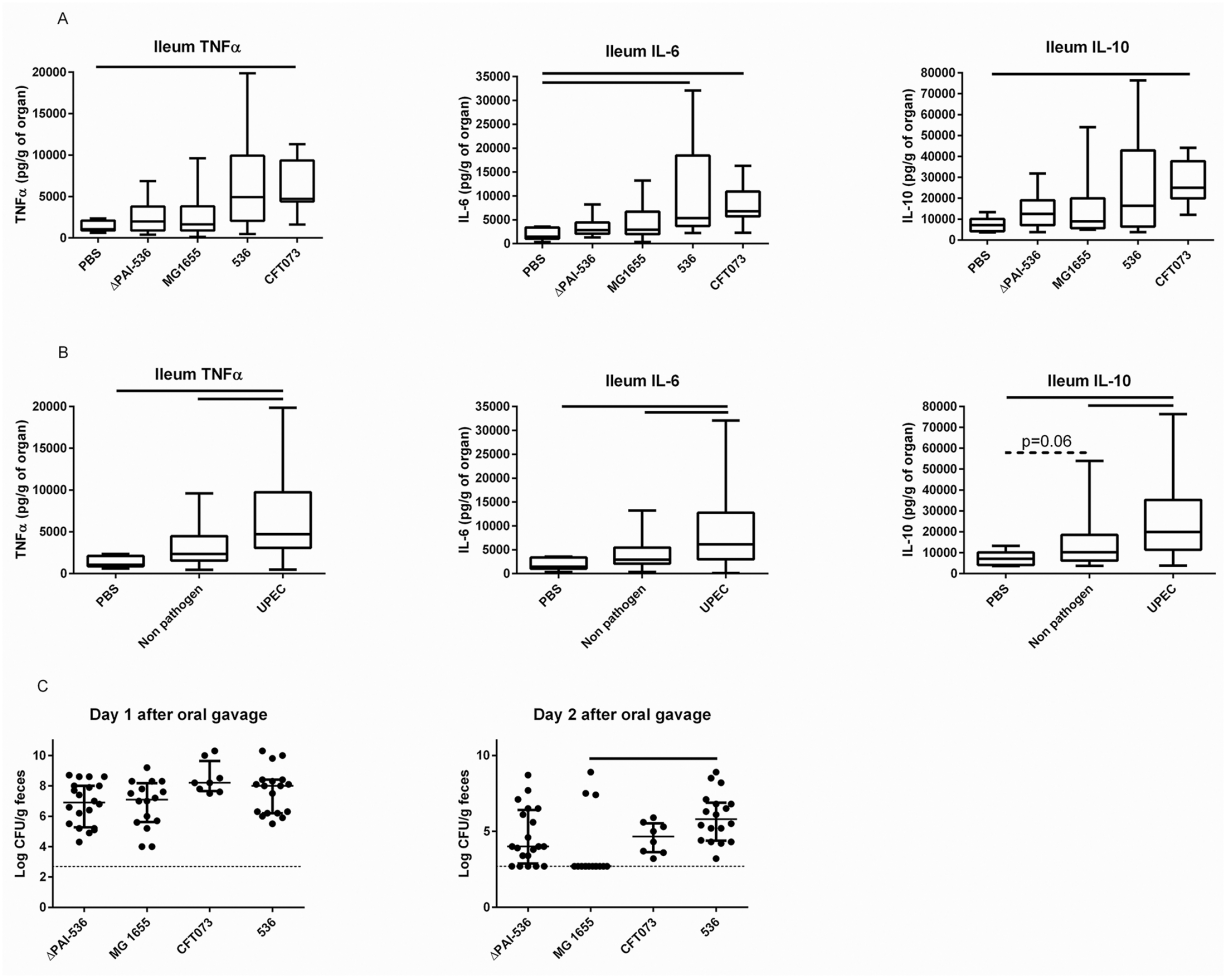
A: TNFα, IL-6, and IL-10 cytokine ELISA measurements in the ileum 2 days after oral gavage with 100 μL of PBS (controls), or 10^7^ CFU of ΔPAI-536, MG1655, CFT073 or *E*. *coli* strain 536. Box and whiskers represent medians and interquartile ranges. Lines indicate significant differences (p<0.05 by Kruskal-Wallis test with Dunn’s correction for multiple comparisons). B: the same measurements are represented in 3 groups: PBS (controls), non-pathogenic or attenuated strains (pooled data from ΔPAI-536 and MG1655) and UPEC strains (pooled data from CFT073 and *E*. *coli* strain 536). Box and whiskers represent medians and interquartile ranges. Lines indicate significant differences (Mann-Whitney U tests). C: *E*. *coli* fecal concentration one and two days (time of the sacrifice for cytokine measurement) after oral gavage. Bars and whiskers represent medians and interquartile ranges. Mice who cleared *E*. *coli* were assigned a bacterial load of 500 CFU g/feces, i.e. 2.7 log_10_ (CFU/g of feces), which was the limit of detection in our model. The line indicates a significant difference (Kruskal-Wallis tests with Dunn’s correction for multiple comparisons). Enterobacteriaceae-free C3H/HeOuJ mice only were used for this experiment. *E*. *coli* CFUs were enumerated in the feces by serial dilution on MacConkey agar plates and expressed as log_10_ (CFU/g feces). Each dot represents one *E*. *coli* fecal count.

*E*. *coli* strain 536 is recognized as a highly virulent strain in the *E*. *coli* species. Deletion of all 7 pathogenicity islands (PAIs) is required to dramatically reduce its virulence [28]. In order to assess whether the ileal innate immune response observed after 536 oral administration was in relation with its intrinsic virulence, ileal cytokine concentrations were compared after oral gavage of C3H/HeOuJ mice with strain ΔPAI-536, which is an isogenic mutant of *E*. *coli* strain 536 in which all 7 PAIs have been deleted[27], and whose pathogenicity is drastically attenuated, non-pathogenic strain MG1655, and two UPEC strains (CFT073 and *E*. *coli* strain 536). Two days after oral challenge, IL-6 concentrations were increased in mice challenged with 536 (positive control) and CFT073 compared to the mice that received PBS (Fig 4A; Kruskal-Wallis test with Dunn’s correction for multiple comparisons, p = 0.005). In addition, IL-10 and TNFα concentrations were higher after oral gavage with CFT073 than after oral gavage with PBS (Kruskal-Wallis test with Dunn’s correction, p = 0.009 and p = 0.01, respectively). To complete the analysis, the same cytokine concentration measurements were pooled in 3 groups: PBS, non-pathogenic or attenuated strains (MG1655 and ΔPAI-536) and UPECs (*E*. *coli* strain 536 and CFT073). Overall, at day 2, the innate immunity response was higher after oral challenge with UPEC than with non-pathogenic or attenuated strains (p ranging between 0.002 and 0.06 for all the comparisons, Fig 4B). Mice who received non-pathogenic or attenuated *E*. *coli* strains tended to have a higher IL-10 ileal production than control mice.

Interestingly, while all mice showed a high intestinal colonization as measured by *E*. *coli* fecal concentration at day 1 after oral gavage (Fig 4C, no difference between groups of mice), a few mice challenged with ΔPAI-536 and most mice challenged with MG1655 had cleared the *E*. *coli* strain at the time of sacrifice for cytokine measurement (day 2). At day 2, when mice who showed no *E*. *coli* in their feces were assigned an arbitrary *E*. *coli* concentration of 10^2.7^ CFU/g of feces (which corresponded to the limit of detection of our method), the colonization of mice was lower with MG1655 than with *E*. *coli* 536 (p = 0.02, Kruskal-Wallis test with Dunn’s correction for multiple comparisons). The proportion of mice who cleared *E*. *coli* was also higher after oral gavage with MG1655 or Δ-PAI536 than after oral gavage with *E*. *coli* strain 536 or CFT073 (p<10^−6^ for the global comparison, and p<10^−3^ for comparisons 2 by 2 by Χ² tests). However, no correlation between *E*. *coli* fecal concentrations and cytokine levels were found.

## Discussion

The aim of this work was to study the integration of a UPEC strain into the digestive tract of a murine host as it models the first step in an extra-intestinal infection. We also wanted to assess whether host detection was modified by the virulent status of the strain.

Most of the previously published models of *E*. *coli* intestinal colonization include antibiotic pretreatment [27, 32, 33]. However, we have shown that it is possible to colonize mice efficiently with various *E*. *coli* strains without the use of antibiotics, at least in the short term. Antibiotics are often useful to increase colonization yield or to reinforce virulence of intestinal pathogens [32], but are not necessary when colonization is the goal. Indeed, in streptomycin-treated mice orally inoculated with *E*. *coli* strain 536, its fecal concentration was between 10^9^ and 10^10^ CFU/g of feces [27], a non-physiological level for commensal *E*. *coli* [15]. Avoiding the use of antibiotics is of value in studying pathogen colonization as antibiotic pretreatment modifies host gut microbiota [34], therefore impacting any subsequent analysis of pathogen-microbiota specific interactions. Furthermore, antibiotic-induced alterations of microbiota drastically affect host immunity [21, 22, 35], another limiting step in many pathogen-host interaction studies. An alternative method has been recently proposed where pregnant female rats were treated with streptomycin and inoculated with streptomycin-resistant *E*. *coli*. The commensal *E*. *coli* strains are transmitted to the offspring and persistently colonized the gut at a level of 10^6^ CFU/g of feces after weaning without any antibiotic [36]. The model that we describe here is simpler, and could be used to study the gut as a reservoir of ExPEC.

*E*. *coli* strain 536 is a known human extra-intestinal pathogen [23] and its high intrinsic virulence has been established in various animal models [26, 28], as well as by the description of its many virulence factors [26]. We have shown that *E*. *coli* strain 536 elicited a transient innate immunity response in the ileum 48 hours after oral administration. However, intestinal histology of colonized mice at 48 hours post oral challenge was normal, and *E*. *coli* 536 fecal colonization did not drop. In addition, we have shown that CFT073, another UPEC strain, also elicited a transient innate immunity response in the ileum. To the contrary, non-pathogenic strain MG1655 and an isogenic mutant of 536 which lacked all 7 PAIs elicited a lower inflammatory response than what was triggered by UPEC strains. Only ileal IL-10 production tended to be higher in mice who received a non-pathogenic or attenuated *E*. *coli* strain than in control mice. Of importance, IL-6 ileal synthesis was not significantly higher after oral administration of ΔPAI-536 than after gavage with PBS, while in contrast it was higher after oral gavage with *E*. *coli* 536 than in control mice. This is a direct demonstration that virulence factors condition the host’s response.

The 7 PAIs of *E*. *coli* strain 536 [26] and the 9 PAIs of CFT073 [37, 38] contain many virulence factors, some of which could constitute “Pathogen Associated Molecular Patterns” (MAMPs) and be recognized by the innate immunity machinery. For example, capsule antigens (*kps*_*K15*_ harbored on PAI V_536_ [39] or *kps*_*K12*_ harbored on PAI I_CFT073_ [38]) or Antigen 43 [40] (PAI III_536_ [26] and PAI I_CFT073_ [38]) are hypothetical candidates for this role. Furthermore, we showed that non-pathogenic strains Δ-PAI536 and MG1655 were inefficient colonizators of the digestive tract compared to ExPEC strains 536 and CFT073. Again, some virulence factors contained on PAIs could contribute to this observation. In particular, adhesion molecules such as fimbriae of type P (PAI II_536_ [26], PAI I_CFT073_ [41], and PAI II_CFT073_ [42]), S (PAI III_536_ [26]), F17 (PAI I_536_ [39]), F1C (PAI-SerX_CFT073_ [38]) and CS12 (PAI I_536_ [39]) could be involved. Consistently, it has been demonstrated that wild-type *E*. *coli* strain 536 was able to outcompete ΔPAI-536 in the streptomycin mouse model of intestinal colonization [27]. Being an ExPEC, i.e. belonging to the B2 phylogroup and possessing numerous virulence factors, may allow better intestinal colonization, based on epidemiological [43, 44], and experimental [27, 45–47] data. This implies that depending on the environment, the same molecule could act as a fitness element (for example when colonizing the digestive tract) or a virulence factor (for example, when colonizing the urinary tract [37]). Pathogenicity appears to be a by-product of commensalism[48].

Our work is an illustration of the physiological role of inflammation in detecting and controlling an incoming potential pathogen. There is abundant literature about the deleterious effects of inflammation in the context of disease. Inflammation leads to fibrotic scars, which can interfere with normal functioning of the affected organ. In extreme cases, excessive inflammation can lead to host death, such as the cytokine storm which occurs in septic shock [49]. This is also the reason why there is a danger in over responding to a pathogen [50]. Conversely, what was observed was likely the response of a normally functioning host. While the host seems to be “blind” to many gut bacteria [51] (here MG1655 and ΔPAI-536 merely triggers cytokine secretion), UPEC strains 536 and CFT073 represent a threat and are therefore detected as such in the ileum. However, activation of innate immunity did not result in a decrease of strain 536 fecal abundance nor result in any visible disease in the host. This observation is an experimental demonstration of the opportunistic nature of ExPEC. Whether the healthy carrier mice have an increased risk for subsequent extra-intestinal infection is not known. This key question is difficult to explore as no model of spontaneous extra-intestinal infection with a high incidence currently exists. Similarly, in human medicine prospective studies on urinary tract infections are lacking. The exact kinetics between the intestinal acquisition of a UPEC strain and the development of a UTI, as well as the triggering factors are unknown.

## Conclusion

Extra-intestinal virulence appears to be an extremely complex phenomenon, and is the result of a network of interactions. Most of the scientific effort has been focused on the sequence of gene activations controlled by the host and/or the pathogen during the infectious processes. Upstream phenomena, such as host colonization by potential pathogens should also be explored as this could open the door to new preventive strategies.
